# Annotated genome sequence of a fast-growing diploid clone of red alder (*Alnus rubra* Bong.)

**DOI:** 10.1093/g3journal/jkad060

**Published:** 2023-03-26

**Authors:** Kim K Hixson, Diego A Fajardo, Nicholas P Devitt, Johnny A Sena, Michael A Costa, Qingyan Meng, Clarissa Boschiero, Patrick Xuechun Zhao, Eric J Baack, Vanessa L Paurus, Laurence B Davin, Norman G Lewis, Callum J Bell

**Affiliations:** Institute of Biological Chemistry, Washington State University (WSU), Pullman, WA 99164, USA; Environmental Molecular Sciences Laboratory, Pacific Northwest National Laboratory (PNNL), Richland, WA 99352, USA; National Center for Genome Resources (NCGR), Santa Fe, NM 87505, USA; National Center for Genome Resources (NCGR), Santa Fe, NM 87505, USA; National Center for Genome Resources (NCGR), Santa Fe, NM 87505, USA; Institute of Biological Chemistry, Washington State University (WSU), Pullman, WA 99164, USA; Institute of Biological Chemistry, Washington State University (WSU), Pullman, WA 99164, USA; Noble Research Institute, Ardmore, OK 73401, USA; Noble Research Institute, Ardmore, OK 73401, USA; Biology Department, Luther College, Decorah, IA 52101, USA; Biological Science Division, Pacific Northwest National Laboratory (PNNL), Richland, WA 99352, USA; Institute of Biological Chemistry, Washington State University (WSU), Pullman, WA 99164, USA; Institute of Biological Chemistry, Washington State University (WSU), Pullman, WA 99164, USA; National Center for Genome Resources (NCGR), Santa Fe, NM 87505, USA

**Keywords:** *Alnus rubra*, red alder, genome, actinorhizal plant, nitrogen fixation

## Abstract

Red alder (*Alnus rubra* Bong.) is an ecologically significant and important fast-growing commercial tree species native to western coastal and riparian regions of North America, having highly desirable wood, pigment, and medicinal properties. We have sequenced the genome of a rapidly growing clone. The assembly is nearly complete, containing the full complement of expected genes. This supports our objectives of identifying and studying genes and pathways involved in nitrogen-fixing symbiosis and those related to secondary metabolites that underlie red alder's many interesting defense, pigmentation, and wood quality traits. We established that this clone is most likely diploid and identified a set of SNPs that will have utility in future breeding and selection endeavors, as well as in ongoing population studies. We have added a well-characterized genome to others from the order Fagales. In particular, it improves significantly upon the only other published alder genome sequence, that of *Alnus glutinosa*. Our work initiated a detailed comparative analysis of members of the order Fagales and established some similarities with previous reports in this clade, suggesting a biased retention of certain gene functions in the vestiges of an ancient genome duplication when compared with more recent tandem duplications.

## Introduction

Red alder (*Alnus rubra* Bong.) is a tree of pivotal ecological, economic, and cultural importance in the forest ecosystems of western North America. Distributed from Alaska to California, it is found principally on western-facing slopes within a few hundred miles of the coast, with small pockets occurring in Idaho. A pioneer species, red alder establishes rapidly on exposed mineral soil, typically after land disturbances such as logging or flooding. It also grows on so-called marginal lands, which are considered unsuitable for conventional agricultural crops, the sustainable use of which could potentially represent an effective route for expanding the area devoted to growing timber and feedstocks without taking land out of food production. Alders significantly help restore degraded soils, including industrial waste ground. Key to the ability of red alder to improve the soil quality of marginal sites is its symbiotic relationship with the actinobacterium *Frankia*. Together they form nitrogen-fixing root nodules, which support plant growth in nitrogen-deficient environments and contribute to overall soil fertility (Benson and Silvester 1993; Hart *et al*. 1997; Deal and Harrington 2006).

From an economic perspective, red alder is mainly used for timber and paper production. In recent years, the annual market value of red alder has exceeded that of Douglas-fir (http://www.westernhardwood.org/Miscellaneous/GIS_hardwood_inventory_6.pdf). Washington State alone has ∼3.7 million acres of annually harvestable/processed hardwoods, with 90% being red alder. In 2002, in Washington State, red alder accounted for >60% of hardwood standing timber available for commercial harvesting (Deal and Harrington 2006). Additional economic potential of red alder comes from its suitability as a biomass feedstock (Gelfand *et al*. 2013). It grows rapidly, with a wood density of *∼*460 kg/m^3^, as opposed, for example, to 380 kg/m^3^ maximum in poplar, this being demonstrably economically more valuable (https://www.engineeringtoolbox.com/). It can be coppiced as a short rotation crop, growing into very dense groves (50,000 trees/acre; DeBell 1972), and can produce a high level of biomass at 4–33 dry tons/acre annually (Resch 1988) in different soil types. In particular, as a pioneer species, it often thrives in large numbers on poor land. In this regard, red alder also has a potential role to play in buffering climate change by sequestering carbon: it is estimated that 1 acre of new forest can sequester about 2.5 tons of carbon annually. Indeed, young trees assimilate CO_2_ at a rate of ∼6 kg/tree/year, this increasing to ∼22 kg/tree/year at about 10 years of age (https://urbanforestrysouth.org/resources/library/citations/method-for-calculating-carbon-sequestration-by-trees-in-urban-and-suburban-settings-1). Growing trees to sequester carbon is viewed as a viable proposition (Cannell 1999).

This study builds on 23 years of clonal selection, initiated by the forestry company Weyerhaeuser and licensed in 2011 to Washington State University (WSU). Historically, clonal red alder variants were carefully selected from the wild, chosen based on their abilities for exceptional growth in adverse conditions, including their ability to thrive under stress, such as high salt, large temperature fluctuations, drought, and differential water use efficiency. The clone chosen for sequencing has an exceptionally high growth rate. The genome sequence reported here will foster an understanding of the basis of this trait, enable the development of molecular markers upon which to build a coherent tree improvement strategy, and stimulate biochemical and other studies that target diverse traits of interest, such as wood chemistry and quality. Moreover, the natural range of red alder is predicted to become hotter and drier, threatening habitat critical to the viability of this important species. The genome reference will also enable the study of genetic variation across the latitudinal range of red alder, allowing the identification of traits that are resilient to these abiotic stresses, and of their associated variants.

## Methods

### Reference plant material

*Alnus rubra* clone 639 is a rapidly growing clone developed as part of a clonal selection program carried out by the forestry company Weyerhaeuser. Vegetatively propagated plants were grown in a greenhouse on the WSU campus in Pullman, WA, USA. Requests for clone 639 plants can be made per WSU policy by contacting Prof. Norman G. Lewis, Institute of Biological Chemistry, Plant Sciences Building 101D, WSU, Pullman, WA 99164-7411, USA.

### DNA preparation

#### Washington State University (WSU)

DNA was prepared from newly emerged 1–2″ long apical leaf samples obtained during mid-afternoon hours in May (2017) from 1-year-old greenhouse-grown saplings maintained under environmental conditions of 15 h of 1,000 watt high-pressure sodium lamps, 24°C and 47% relative humidity. The leaves were flash-frozen in liquid nitrogen and stored at −80°C until processed. Frozen leaves were ground to a fine powder in liquid nitrogen using a mortar and pestle. Genomic DNA was isolated from leaves using a DNeasy Plant Mini Kit (Qiagen). Fifty milligrams of the pulverized sample were suspended in Qiagen Buffer AP1 (400 mL) containing β-mercaptoethanol (10 µL). Following incubation with RNase A (400 µg) in a 65°C water bath for 30 min, samples were processed according to the DNeasy Plant Handbook protocol. Purified DNA was eluted from the DNeasy Mini spin columns in Qiagen AE elution buffer (70 μL).

#### Pacific Northwest National Laboratory (PNNL)

DNA was extracted from fully expanded leaves of 1-year-old greenhouse grown seedlings in early summer (2017 May 10). The greenhouse was located at the WSU Tri-Cities campus in Richland, WA. The leaves were quickly cut from the trees, wrapped in damp paper towels, and cooled overnight at 4°C. A Joint Genome Institute plant nuclear DNA protocol was used, consisting of incubation in guanidine-HCl/proteinase K lysis buffer, Qiagen Genomic-tip purification, and isopropanol precipitation (https://jgi.doe.gov/user-programs/pmo-overview/protocols-sample-preparation-information/). Qiagen Genomic-tips (100/G) were used according to the manufacturer's instructions. DNA was suspended in ∼200 µL EtOH solution and stored at −80°C until ready for use.

### PacBio sequencing

#### National Center for Genome Resources (NCGR)

20-kb PacBio libraries were prepared from leaf tissue DNA. The following kits were used according to the manufacturer's instructions: SMRTbell Template Prep Kit 1.0 (catalog number 100-259-100), DNA Sequencing Bundle 4.0 v2 (catalog number 100-676-400), DNA/polymerase-binding kit P6v2 (catalog number 100-372-700), MagBead Kit v2 (catalog number 100-676-500), and DNA Internal Control Complex P6 (catalog number 100-356-500). Libraries were loaded onto SMRT cells and sequenced on a PacBio RSII instrument using P6 polymerase C4 chemistry with 6 h movie times.

#### Pacific Northwest National Laboratory (PNNL)

Leaf DNA was sheared to 10–20 kb using a Covaris g-Tube, concentrated using AMPure PB magnetic beads, and quality was evaluated using the Qubit dsDNA HS Assay Kit and with the Sage Science Pippin Pulse Electrophoresis system. Size selection was done using the BluePippin size-selection system. These were then used to generate PacBio long-read libraries for sequencing. Primer annealing and polymerase-binding reactions were prepared using the Binding Calculator from PacBio, these being based on available sample volume, concentration, and insert size using default settings. PacBio RSII sequencing was performed with 6 h movies at Yale Center for Genome Analysis.

HDF5, FASTA, and fastq files from both sequencing operations were used for combined analysis at NCGR. The sequence data represent ∼93× genome coverage (presuming an initial genome size of 0.5 Gb, based on other *Alnus* sp. entries in the Kew Gardens *c*-values database; Garcia *et al*. 2014).

### Illumina sequencing

Leaf DNA as prepared above (PNNL) was sent to Lucigen Inc. (Middleton, WI, USA), where it was evaluated by agarose gel electrophoresis for RNA contamination and integrity. Having passed both quality checks, a paired-end Illumina TruSeq DNA library was constructed, and the concentration was determined using a Qubit fluorometer (Thermo Fisher). The insert size was estimated by Agilent Bioanalyzer to be 676 bp. The library was sent to the Genomics Service Center at WSU, Spokane, where it was sequenced in paired-end 250 bp configuration on an Illumina HiSeq 2500 instrument. The data were demultiplexed and trimmed by the center before being delivered in FASTQ format.

### Genome assembly and annotation

Sequence data were assembled with FALCON (https://github.com/PacificBiosciences/FALCON), while assembly, polishing, and correction were completed using Daligner (https://github.com/cschin/DALIGNER) and Quiver (Chin *et al*. 2013). Completeness of assembly, compared with other members of the Fagales (Supplementary Table 1), was evaluated using Benchmarking Universal Single-Copy Orthologs (BUSCO) software version 4.01 (Manni *et al.* 2001) and the eudicots_odb10 lineage data set, containing 31 species and 2,326 BUSCOs. Gene annotation was performed using the MAKER-P pipeline (Cantarel *et al*. 2007). The first step included generation of a masked Clone 639 genome from repetitive elements and transposable element proteins using Repeatmasker (Tarailo-Graovac and Chen 2009) and Repeatrunner (Smith *et al*. 2007), respectively. Annotation included *ab initio* gene predictions using RNA-seq data as species-specific evidence, publicly available ESTs from the order Fagales (including *Alnus glutinosa*, *Betula pendula*, and *Quercus* spp.) as close relatives, and *Arabidopsis thaliana* as a model species to train the gene prediction software SNAP (Korf 2004) and AUGUSTUS (Stanke *et al*. 2004). Transfer RNAs were identified and annotated by using tRNAscan-SE (Lowe and Eddy 1997). False positives were discarded by filtering transcripts by their Annotation Edit Distance (AED) and protein homology by running InterProScan (Zdobnov and Apweiler 2001). Sequence repeat annotations were performed by running Repeatmasker, RepeatModeler (Flynn *et al*. 2020), TransposonsPSI (http://transposonpsi.sourceforge.net/), and LTRharvest (Ellinghaus *et al*. 2008). Repeats were classified using the MIPS/PGSB Repeat Element Database (Spannagl *et al.* 2017), as well as TransposonPSI and RepeatModeler.

### Flow cytometry

Genome size was estimated by flow cytometry using nuclei from red alder and tomato (*Lycopersicon esculentum* L.), a standard with a well-known genome size (Doležel *et al*. 1992). Samples from unexpanded red alder leaves kept at 4°C were chopped with a razor blade together with freshly harvested tomato leaves. Cold chopping buffer (15 mM HEPES, 1 mM EDTA, 80 mM KCl, 20 mM NaCl, 300 mM sucrose, 0.2% Triton X-100, 0.1% DTT, 0.5 mM spermine, and 0.25 mM polyvinylpyrolidone-40; modified from Dart *et al*. 2004) was added to the leaves prior to chopping, then again before filtering. Once filtered through 2 layers of Miracloth (Millipore Sigma) into a 1.5-mL microtube, the sample was centrifuged at 1,000 × g for 5 min. After removing the supernatant, the pellet was resuspended in 45 μL of 1.68 mM propidium iodide and 0.955 mL of a solution containing 100 mL MgSO_4_ buffer, 100 mg DTT, and 2.5 mL Triton X100. The MgSO_4_ buffer consisted of 0.25 g MgSO_4_·7 H_2_O, 0.37 g KCl, and 0.12 g of HEPES dissolved in 100 mL H_2_O with the pH adjusted to 8.0 (Arumuganathan and Earle 1991). The sample was then analyzed using a FACScan Flow Cytometer (BD Biosciences, San Jose, CA, USA). Peak positions corresponding to both sample and standard were recorded and the *c*-value for the red alder sample was computed.

### Small secreted peptide identification

To identify genes encoding small secreted peptides (SSPs), a bioinformatics pipeline was applied that was recently used for *Medicago truncatula* (de Bang *et al*. 2017; Boschiero *et al*. 2019). First, the SPADA package (Zhou *et al*. 2013) was used to identify short peptide-coding genes in the red alder Hidden Markov Models (HMMs) from *M. truncatula* and HMMs from the PlantSSPdb (Ghorbani *et al*. 2015). New genes identified by SPADA were integrated with general protein gene annotations, redundant genes being removed. Next, the Plant SSP Prediction Tool available at https://mtsspdb.zhaolab.org/database/ was applied to the red alder protein annotations. This applies different approaches to identify SSPs, such as the presence of signal peptide cleavage sites by SignalP server (Petersen *et al*. 2011), homologies with previously identified known SSPs, protein size, and transmembrane (TM) helix prediction. The combined predictions classify SSPs as “known,” “likely known,” or “putative.” A known SSP has a protein length of ≤200 amino acids, SignalP *D*-score of >0.25, and homology with previous SSPs, while a putative SSP has a protein length of ≤230 amino acids, SignalP *D*-score of >0.45, no TM domains, and no significant homologies with known SSPs. A likely known SSP has significant homologies to known SSPs and a small protein length (≤250 amino acids).

### K-mer analysis

KAT version 2.4.1 (Mapleson *et al*. 2017) was applied to 83 Gb of paired-end 250 bp Illumina reads and a FASTA file of the genome assembly. The KAT comp program was run with -m equal to 21, 31, 41, and 51. K-mer spectra were derived from output files using the program kat plot spectra-cn. To prepare input for GenomeScope, k-mers were counted with jellyfish version 2.2.10 using the command jellyfish count -C -m k (21,31,41,51) -s 1000000000 -t 32 and jellyfish histo, respectively. Resulting .histo files were used as input to GenomeScope 2.0 (Ranallo-Benavidez *et al*. 2020) parameterized as diploid or tetraploid (-p 2 or -p 4), running under R version 3.6.0.

### Tandem repeat analysis

To identify tandemly repeated DNA, tandem repeats finder (TRF; Benson 1999) was applied to a collection of PacBio reads in which the polymerase sequenced the template at least 5 times. These reads were generally of high quality. The output of TRF was parsed with a Perl script to extract the repeat features and to write out the repeat monomers in the 50–500 bp range as a FASTA file. Repeats in the most abundant 176–182 bp category were studied further, by comparing them pairwise with one another using BLASTN. Pairs of similar repeats, having an E-value of <1e−10, were grouped together into repeat classes. Arrays of tandem repeats in each PacBio read were curated by hand and split into sets of monomers at a motif common to the borders of all repeats (AGTTTT). Each set of monomers was aligned with Clustal Omega (Sievers and Higgins 2021) and the consensus extracted with the cons program of the EMBOSS package (Rice *et al*. 2000). Consensus repeat monomers from each PacBio read were aligned with each other, and similarity trees were generated using the software package Seqotron (Fourment and Holmes 2016). The same approach was applied to the *A. glutinosa* genome assembly (NCBI assembly accession GCA_003254965.1) and *B. pendula* PacBio reads (SRA accession ERR2003767.1) in order to compare tandem repeats among these related species.

### Gene duplication analysis

Protein representations of gene annotations were aligned to one another using BLASTP 2.9.0 with the following parameters: -num_threads 32 -evalue 1e-10 -max_target_seqs 5 -outfmt 6. The output table and the annotations GFF file were used as input to MCScanX (Wang *et al*. 2012). Output files of MCScanX were parsed in a Perl script to identify start and end coordinates of each duplicate segment and to identify genes therein. Tandemly repeated genes were also evaluated with MCScanX. Protein representations of duplicated genes were aligned to *A. thaliana* proteins (https://www.arabidopsis.org) using BLASTP. Hits with E-values <1e−10 were analyzed in GOATOOLS (Klopfenstein *et al*. 2018), to identify gene ontology (GO) terms significantly enriched at *P* < 0.05 by Fisher's exact test. Further GO-term annotation was done with eggNOG-Mapper (Huerta-Cepas *et al*. 2017). All red alder annotated proteins and the subsets contained in the self-syntenic and tandemly repeated fractions were submitted to the eggNOG-Mapper server (http://eggnog-mapper.embl.de/). To evaluate the genome nucleotide-level repetitive content, the genome assembly was compared with itself using MUMmer (Delcher *et al*. 2003). Alignments >30 bp with at least 95% identity were included. Annotated protein-coding genes of 7 other species from the order Fagales were obtained from NCBI and analyzed with red alder protein annotations using OrthoFinder 2.3.3 (Emms and Kelly 2019; Supplementary Table 1). The main use of the OrthoFinder output was to collect genes into paralogous groups for *K*_s_ estimation. Nonsynonymous substitution rates (*K*_s_) were computed using a Perl script employing BioPerl modules, in particular Bio::Align::DNAStatistics, which computes *K*_s_ using the Nei–Gojobori algorithm (Nei and Kumar 2000).

### SNP discovery

Eighty-three giga base pairs of paired-end 250 bp genomic DNA Illumina reads were aligned to the haploid genome assembly with HISAT2 (Kim *et al.* 2019) with parameters: --no-spliced-alignment--threads 32 --no-unal. Duplicate BAM file reads were marked using sambamba (https://lomereiter.github.io/sambamba/docs/sambamba-markdup.html) and variant calling was done with FreeBayes (Garrison and Marth 2012) using 2 parameterizations: -P 0.001 -p 2 -i -X -u -C 10 -m 30 -q 20 -! 30, and -P 0.001 -p 2 -i -X -u -C 5 -m 30 -q 20 -! 10. The resulting SNP calls were used to evaluate the quality of the genome assembly by running the BCFtools (Danecek *et al*. 2021) command: bcftools stats --samples on the VCF files. The PSC section of each statistics report was consulted to determine the number of homozygous reference and homozygous nonreference SNP calls, which should both be low if the assembly quality is high.

## Results and discussion

**Fig. 1.**
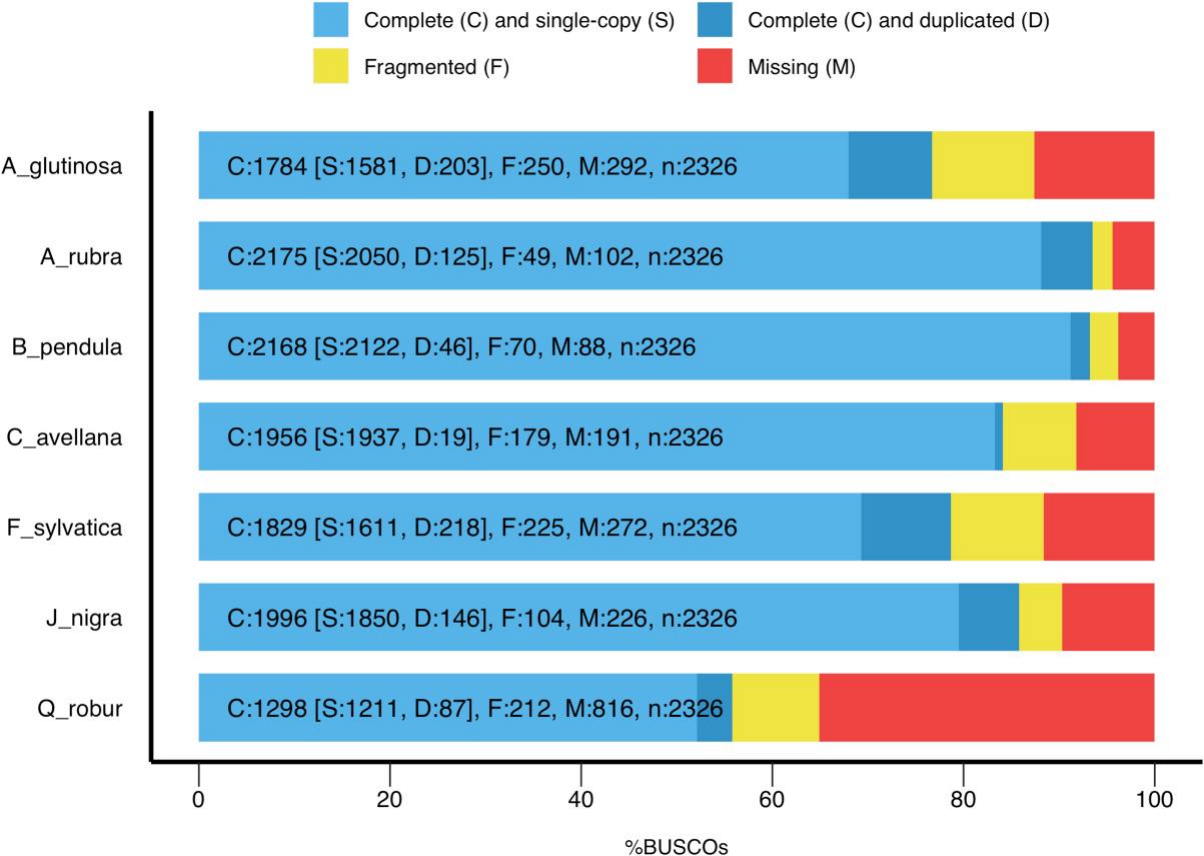
Assembly completeness assessment with BUSCO for *Alnus rubra* vs select members of the order Fagales.

Genome assembly statistics are in Table 1. The primary assembly was treated as a typical haploid, as commonly used for annotation and genome size estimation. The associated contigs are probably sequences allelic to primary contigs, useful for variant discovery and evaluating allelic expression differences. Overall genome GC composition was estimated to be 37%. Genome assembly completeness was assessed using Benchmarking Universal Single-Copy Orthologs (BUSCO v.4.01; Manni *et al*. 2021), a quantitative assessment of genome assembly and annotation completeness based on evolutionarily informed expectations of gene content across lineages. BUSCO analysis using the eudicot lineage revealed that 2,050 (88.13%) of 2,326 total single-copy ortholog genes were present in the *A. rubra* genome assembly and an additional 125 (5.37%) genes were duplicated, resulting in a completeness estimate of 93.5%. This genome completeness is comparable or superior to that of several other genomes in the Fagales available at the time this research was conducted (Fig. 1), including additional members of the Betulaceae, such as silver birch (*B. pendula*), black alder (*A. glutinosa*), and European hazelnut (*Corylus avellana* L.); the Fagaceae including European beech (*Fagus sylvatica*) and English oak (*Quercus robur*); and the Juglandaceae, such as black walnut (*Juglans nigra*) with completeness estimates of 93.2, 76.7, 84.1, 78.7, 55.8, and 85.8%, respectively (Fig. 1). In support of the BUSCO analysis of conserved single copy genes, we evaluated the representation of an assembled transcriptome in the genome assembly. About 92.4% of the assembled transcripts were found in the genome assembly (not shown).

**Table 1.** Red alder genome assembly statistics, including the complete assembly (primary and associated contigs), and primary contigs only.

	Primary contigs	Primary and associated contigs
Number of contigs	1,363	2,717
Contig N50	1.74 Mb	1.53 Mb
Longest contig	8.25 Mb	8.25 Mb
Mean contig length	0.356 Mb	0.204 Mb
Assembly length	485.60 Mb	552.90 Mb
GC content	36.83%	36.70%

Associated contigs are from the regions of the assembly graph where there was sufficient variation to infer the haplotypes of primary contigs.

### Genome annotation

A total of 52,758 protein coding genes were predicted after filtering using an AED of <1, and comparison with known protein domains. Because of our interest in nitrogen-fixing symbiosis, further annotation focused specifically on SSPs, a signaling molecule class that participates in a vast range of plant growth and development processes, including root development and nodulation (Djordjevic *et al*. 2015; Kereszt *et al*. 2018). A total of 2,494 of the genes predicted by the MAKER pipeline were identified as SSPs. Additionally, 1,043 new SSP genes were predicted, bringing the total number of genes to 53,801, within the predicted ranges of other Fagales members (Supplementary Table 1). Supplementary Table 3 shows all of the predicted SSPs, along with their annotation details and whether they are classified as already known SSP (311), likely SSP (219), or putative SSP (1,968).

### Repetitive DNA content

Annotation of transposable elements used RepeatMasker (Tarailo-Graovac and Chen 2009), TransposonsPSI (http://transposonpsi.sourceforge.net/), and LTRharvest (Ellinghaus *et al*. 2008), with the MIPS/PGSB Repeat Element Database applied for their classification (Spannagl *et al.* 2017). The estimated genome total repeat content was 130.5 Mb, averaged from different approaches employed, representing 23–31% (lower and upper size estimates) of the genome. Gypsy and Copia LTR retrotransposons were the most abundant repeats, occupying 3.5 and 4% of the genome (Supplementary Table 2). Equivalent proportions in silver birch, the closest relative with data available, were 8.5 and 2.3%, respectively (Salojärvi *et al*. 2017).

Another class of repetitive DNA consists of tandemly repeated units. These can be associated with important functional elements of the chromosome, such as centromeres (Melters *et al*. 2013). To identify tandemly repeated DNA, we selected a subset of 15,433 high-accuracy PacBio reads having at least 5 circular consensus reads. Tandemly repeated DNA arrays were identified by applying TRF (Benson 1999). The most common repeat unit identified was ∼180 bp long, typical of centromeric DNA (Melters *et al*. 2013), found in 43 PacBio reads. Mapping this repeat back to the haploid assembly showed that it tends to be found in contiguous arrays; DNA segments with at least 95% repetitive content averaged 8,795 bp. These arrays were found concentrated in smaller contigs with a median size of 9,614 bp. The longest array observed was 59 kb (Supplementary Table 4). A comparative approach was applied to the genomes of *A. rubra*, *B. pendula*, and *A. glutinosa*. Red alder and *B. pendula* tandem repeats revealed no similarities. The major class of red alder tandem repeats was, however, closely related to a small family of repeats of similar length in *A. glutinosa*. The relationships among the repeat consensus units are shown in a neighbor-joining tree (Supplementary Fig. 1). The consensus sequences of these repeats, which are viable candidates for centromeric DNA, are in Supplementary Table 5.

### Ploidy and genome size

**Fig. 2.**
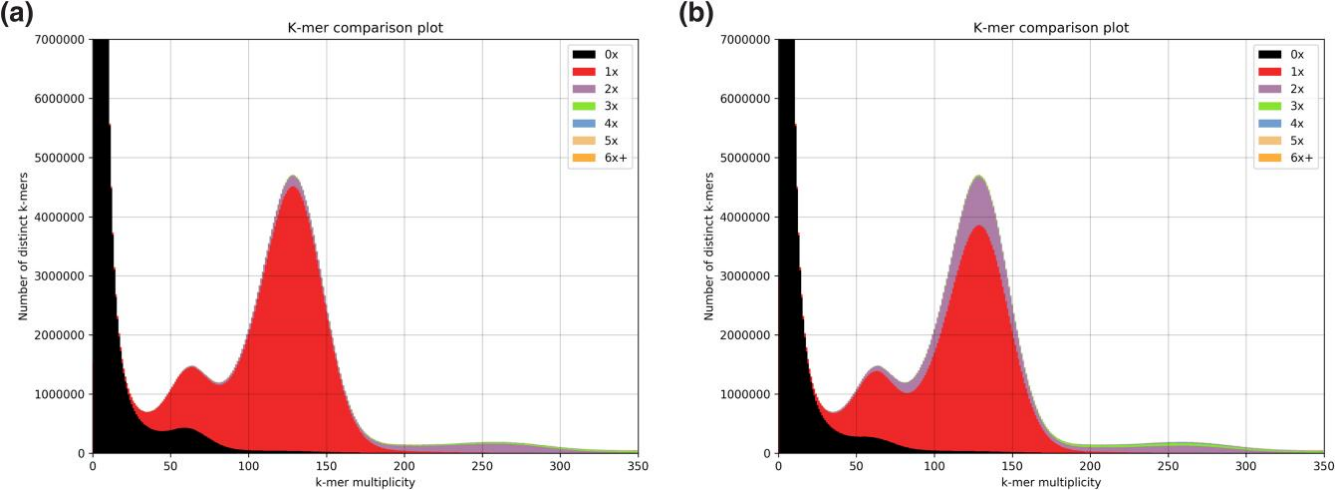
K-mer analysis toolkit (KAT) analysis of 21-mers. a) Primary (haploid) genome assembly. b) Primary contigs plus associated contigs.

Loveless (2021) used molecular genetic methods, in some cases confirmed by microscopy, to show that red alder diploids and tetraploids were present in 9 out of 10 sampling locations in Washington, Oregon, and Idaho. We explored the ploidy of Clone 639 using k-mer analysis. K-mer spectra (*k* = 21) from analysis with the k-mer analysis toolkit (Mapleson *et al*. 2017) are shown in Fig. 2. The associated contigs contributed most of the duplicated k-mers in the genome, i.e. those mapping twice to the reference genome (purple shading), further evidence that associated contigs represent allelic segments of primary contigs. The k-mer multiplicities of 65 and 130 (Fig. 2) represent heterozygous and homozygous portions of the genome assembly. This analysis strongly suggests that our red alder clone is diploid, although autotetraploidy cannot be ruled out. Analysis of the primary assembly with *k* = 31, 41, and 51 did not differ appreciably from *k* = 21 (Supplementary Fig. 2). In support of diploidy, red alder k-mers were also analyzed using GenomeScope 2.0 (Ranallo-Benavidez *et al*. 2020), parameterized as diploid or tetraploid (Supplementary Fig. 3). The data fitted the GenomeScope diploid models closely at all values of *k*, whereas the data deviated from the tetraploid models.

Five sources of data were used to estimate the genome size to be in the range 415–563 Mb. (1) The primary contig assembly has a total length of ∼486 Mb. (2) Our flow cytometry analysis indicated 2C = 1.14 pg/nucleus which provides a genome size estimate of 563 Mb. Three samples yielded the same flow cytometry estimate. (3) GenomeScope estimated the genome size at 414 Mb (*k* = 21) to 423 Mb (*k* = 51). (4) Extrapolating read coverage of single copy BUSCO genes to the haploid assembly gives a genome size estimate of 452 Mb. (5) The findGSE algorithm (Sun *et al*. 2018) provided estimates of 494 (*k* = 21) to 557 (*k* = 51) Mb. These estimates span a wide range of values, but are in the range reported for other alders (Kew Gardens *c*-values database, cited by Garcia *et al*. 2014).

### SNP discovery

**Fig. 3.**
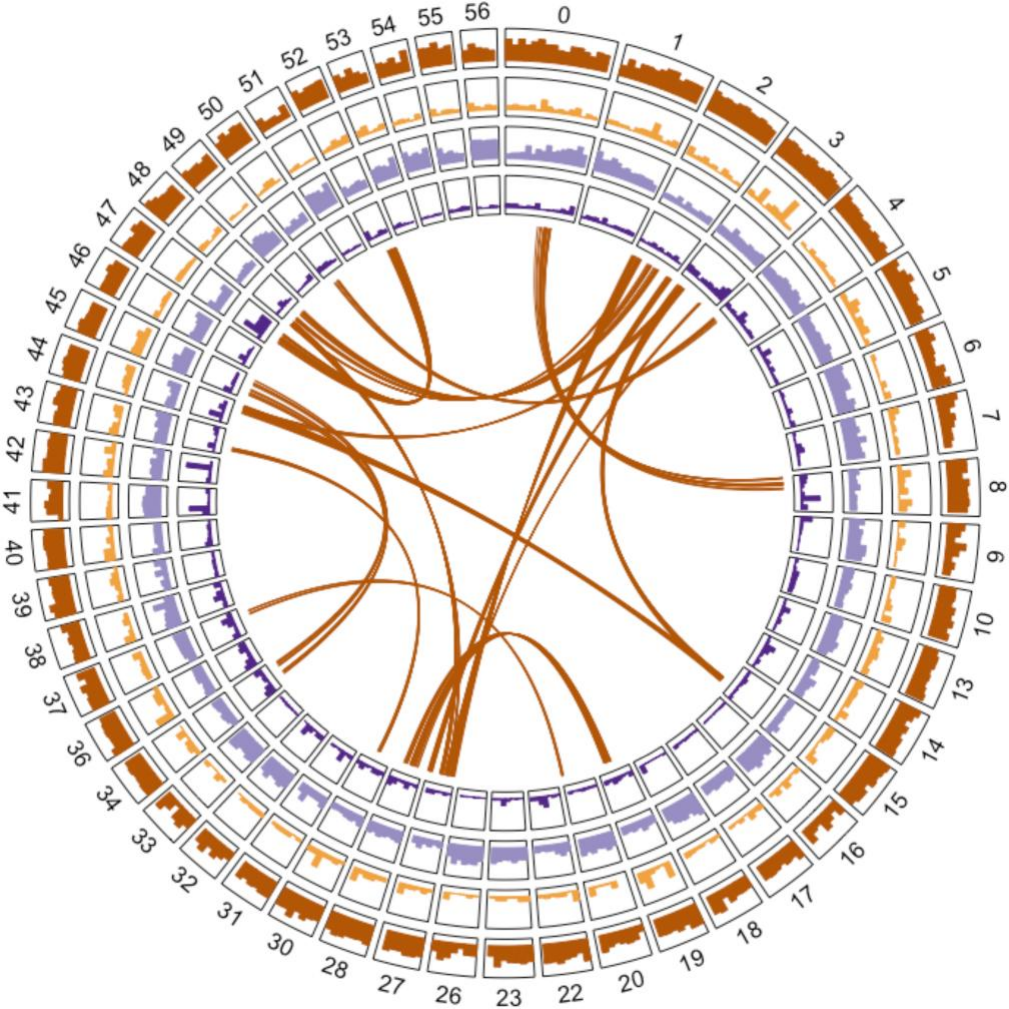
Annotation summary of the fifty largest contigs in the red alder genome. In order from the outside: all protein coding genes, SSP genes, LTR transposons, SNPs, and duplicated segments displayed as ribbons.

Genome heterozygosity was evaluated by aligning Illumina genomic DNA sequence reads from the same clone to the haploid assembly. Paired-end 250 bp Illumina reads (83 Gb total) were aligned to the haploid genome assembly using HISAT2 (Kim *et al*. 2019), with variant calling utilizing FreeBayes (Garrison and Marth 2012). Parameters using a minimum sequencing depth of 30, a minimum of 10 reads for the minor allele, a minimum mapping quality of 20, and a minimum sequence quality of 20, gave 257,020 SNPs. Relaxing the read number having a minor allele to 5 gave 281,163 SNPs. Using the more conservative criteria and the largest genome size estimate (563 Mb), this gave a SNP approximately every 2,600 bp. With more relaxed SNP calling criteria and the smallest genome size estimate (415 Mb), the inter-SNP distance was 1,570 bp. These SNPs are by definition heterozygous and do not address population-level variation. Nonetheless, they will be useful in future selection, breeding, and population studies. The distribution of SNPs in the 50 largest contigs is illustrated in Fig. 3. The BAM file statistics are also supportive of a high-quality assembly; of 172,567,868 read pairs, only 1,355,595 (0.79%) had partners mapping to different contigs with high-quality alignments (mapQ > 5). Because we aligned short reads from the same plant to the haploid assembly, all SNPs detected are expected to be heterozygous. Accordingly, 2 important metrics are the numbers of homozygous reference and homozygous “alt” SNP calls. If the assembly quality is high, both of these should be very low. In the case of the more stringent SNP calling parameters, these numbers were 0 and 607 (of 257,020), respectively. In the case of the more relaxed parameters, they were 0 and 1,304 (of 281,163), respectively. These statistics support the conclusion that the assembly is accurate.

### Gene duplications

Genome complexity was analyzed using MCScanX (Wang *et al*. 2012), which classifies genes into paralogous groups, and identifies tandem gene duplications and duplications present in self-syntenic (collinear) genome segments. This analysis identified 211 duplicated segments averaging 561 kb in length, the largest being 5 Mb and the smallest 5.6 kb. Together, these account for 238 Mb or more than half of the genome (Supplementary Table 6). Duplicated genes per self-syntenic segment ranged from 4 to 41, averaging 9. In total, 1,931 duplicated gene pairs residing in self-syntenic segments were identified. MCScanX also identified 1,220 tandemly repeated gene pairs. To explore potential relationships between duplicated regions and possible evolutionary events, further analysis utilized the dissect_multiple_alignment program of the MCScanX package to estimate gene numbers present in blocks of differing depths. The great majority were present in blocks of depth 1, 2, and 3 (40,205, 10,767, and 3,033 genes, respectively). Duplicated segments in the largest 50 contigs (shown as ribbons in the center of Fig. 3) most likely represent vestiges of the γ whole-genome duplication (WGD) in the eudicot lineage (Vekemans *et al*. 2012), now mostly represented by segment pairs. Very few genes are present in blocks of greater depth. The 76 genes in blocks of depth 4 probably represent tandem duplications within collinear segments. Investigation of the 15 genes in blocks of depth 8 assisted with quality control of the genome assembly, since all were found to be present on contigs containing plastid DNA. Genome self-comparison at the nucleotide level using MUMmer (Delcher *et al*. 2003) compared 30-mers with a threshold of 95% for identity. About 127 Mb (23–30% of the red alder genome, depending on the size estimate) is composed of repeated DNA segments. This is similar to the fraction of genes (26%) present in the combined collinear and tandemly repeated classes.

Prior work (Salojärvi *et al*. 2017) established that in *B. pendula*, syntenically and tandemly duplicated genes were enriched for different GO terms, implying selective retention of transcription factors in syntenic segments, and expansion by tandem duplication of genes involved in secondary metabolism and host defense, among others. That work (Klopfenstein *et al*. 2018) identified GO terms significantly enriched at *P* < 0.05 by Fisher's exact test following correction for multiple testing. We applied the same tool to *Arabidopsis* annotations of our syntenic and tandemly repeated genes (GOATOOLS version 1.0.3). The results, shown in Supplementary Tables 7 and 8, reflect these findings: enriched genes in self-syntenic segments emphasized responses to chemical entities, including hormones, protein kinase activity, and, in particular, transcription factors. Tandemly repeated genes were enriched for GO terms including environmental responses, i.e. wounding, oxidoreductase activity, responses to external stimuli (including other organisms), and secondary metabolism.

Assuming non-neutrality of nonsynonymous nucleotide substitutions, determining the *K*_s_ rate can assist with determining the relative ages of gene duplications (Blanc and Wolfe 2004; Maere *et al*. 2005; Tang *et al*. 2008). We determined the distribution of pairwise gene *K*_s_ values for all paralogous gene pairs and for gene pairs in the collinear and tandemly duplicated sets (Supplementary Fig. 4). Genes remaining from the γ-duplication are most likely to be those in the secondary peak at *K*_s_ of ∼0.8–1.0. The *K*_s_ spectrum of genes present in the self-syntenic regions was consistent with some recent tandem duplication of genes themselves remaining in multiple copies from the γ duplication. *K*_s_ for gene families in other members of the order Fagales was also computed (Supplementary Fig. 5), based on ortho groups as above, with the gene annotation sources for each species indicated in Supplementary Table 1. Gene duplication histories, as deduced from *K*_s_ distributions, were similar for *A. rubra*, *A. glutinosa*, *F. sylvatica*, *Q. robur*, and *Casuarina glauca*. By comparison, *B. pendula* and *C. avellana* retained greater numbers of gene duplications from the γ-WGD, compared with recent tandem duplications. *Juglans nigra*, a sister to the branch containing *Betula*, *Alnus*, *Corylus*, and *Casuarina* (Li *et al*. 2004) contained evidence of a much more recent WGD, as indicated by the large number of gene families with *K*_s_ of ∼0.3. This duplication is absent from other Fagales members we analyzed. In support of this observation, the recent report of the *Juglans regia* genome (Marrano *et al*. 2020) illustrated extensive collinearity of whole chromosomes. Cumulatively, the evidence suggests remnants of the γ duplication in red alder and ongoing tandem gene duplications with deletion of duplicates over time are not under selective constraints (Blanc and Wolfe 2004).

### Conclusion

We report the annotated genome of a rapidly growing clone of *A. rubra*, a tree of significant ecological, cultural and economic importance. Although fragmented, the assembly is as or more complete than other sequenced genomes in the order Fagales available at the time this research was conducted, and the annotated genes, repeats, and variants provide the necessary resources for understanding red alder's many interesting traits, as well as for future breeding and selection endeavors, and population studies. This study initiated comparative genomics analysis of the order Fagales; the addition of the red alder genome to this collection facilitates such work, which is ongoing.

## Supplementary Material

jkad060_Supplementary_Data

## Data Availability

The PacBio and Illumina genomic sequence reads are deposited in the NCBI Sequence Read Archive (SRA) under BioProject ID PRJNA689849. The genome assembly has been deposited at GenBank under the accession JAJPGS000000000. Supplementary files that fully describe the data reported herein have been uploaded to Figshare and can be accessed at https://doi.org/10.6084/m9.figshare.17532155. These include the assembly, annotated genes, proteins and repeats, and VCF files containing SNPS. Supplemental material available at G3 online.
