# Supplementary material for: Annotated genome sequence of a fast-growing diploid clone of red alder (Alnus rubra Bong.)
Source: G3 (Bethesda). 2023 Mar 26;13(6):jkad060. doi: 10.1093/g3journal/jkad060 (PMC10234377; doi:10.1093/g3journal/jkad060)
Supplement: jkad060_Supplementary_Data [file jkad060_supplementary_data.zip › Figures_S1-S5_Legends_G3-2023-404103.docx]

Figure S1. Neighbor Joining tree derived from Seqotron analysis of multiple alignments of consensus sequences of families of tandem repeats. Each label represents the consensus repeat unit from an array of repeats in one PacBio read.

Figure S2. KAT K-mer spectra of paired end 250 bp Illumina reads represented in Clone 639 genome assembly, primary contigs only. A, k = 31. B, k = 41. C, k = 51.

Figure S3. K-mer analysis with GenomeScope 2.0, parameterized as either diploid or tetraploid, with four values of k. A, k = 21, diploid. B, k = 31, diploid. C, k = 41, diploid. D, k = 51, diploid. E, k = 21, tetraploid. F, k = 31, tetraploid. G, k = 41, tetraploid. H, k = 51, tetraploid.

Figure S4. Distributions of K_s_ computed from red alder paralogous gene groups. (a) Computed from randomly chosen pairs of genes in each group. (b) Computed from the mean of each group. (c) Computed for duplicated gene pairs in self-syntenic segments. (d) Computed from tandemly repeated gene pairs.

Figure S5. K_s_ distributions for orthogroups in eight species of the order Fagales.
