# Supplementary figures and images for: Annotated genome sequence of a fast-growing diploid clone of red alder (Alnus rubra Bong.)
Source: G3 (Bethesda). 2023 Mar 26;13(6):jkad060. doi: 10.1093/g3journal/jkad060 (PMC10234377; doi:10.1093/g3journal/jkad060)

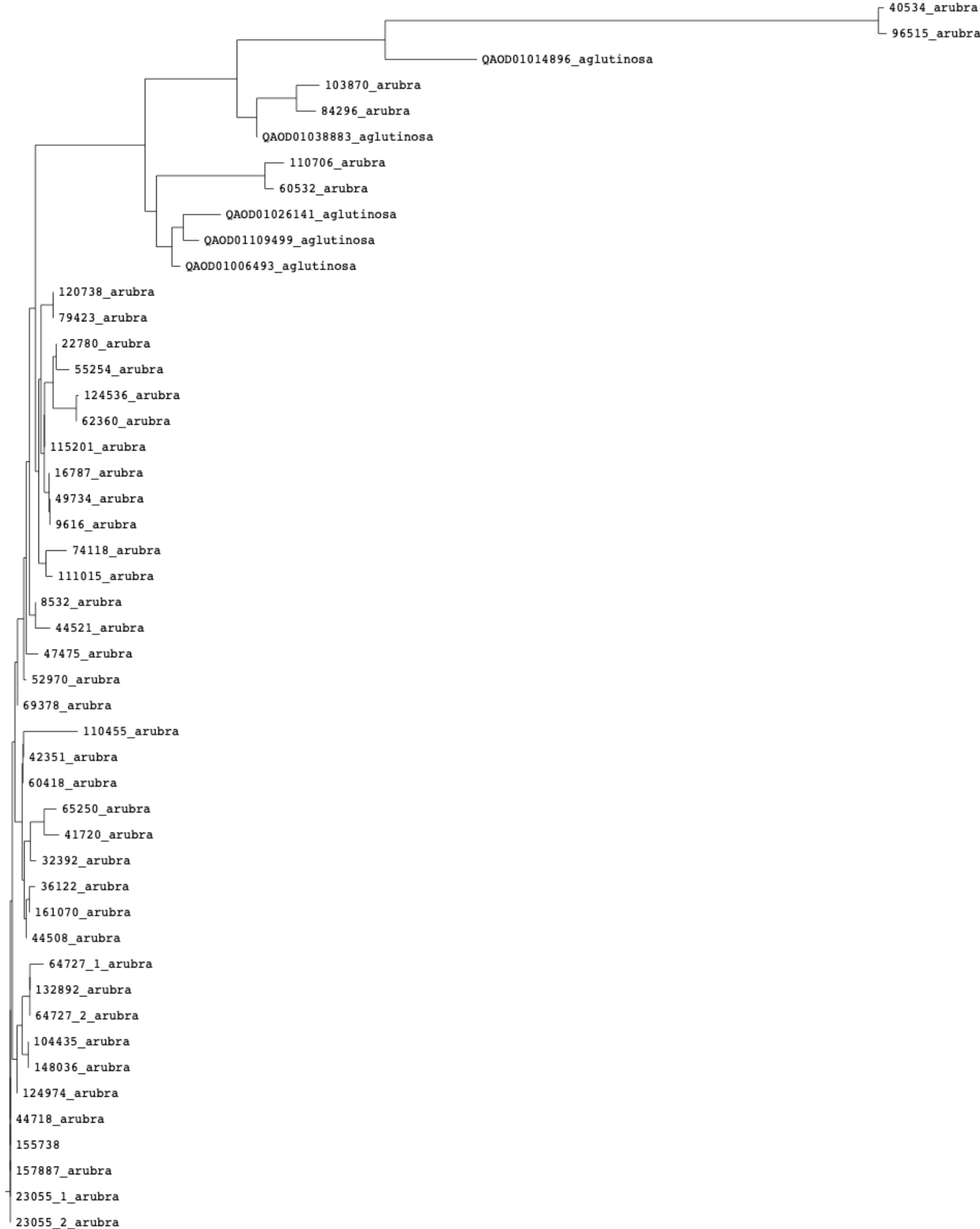

0.0416

Supplement: jkad060_Supplementary_Data [file jkad060_supplementary_data.zip › Figure_S1_G3-2023-404103.pdf]

**A**

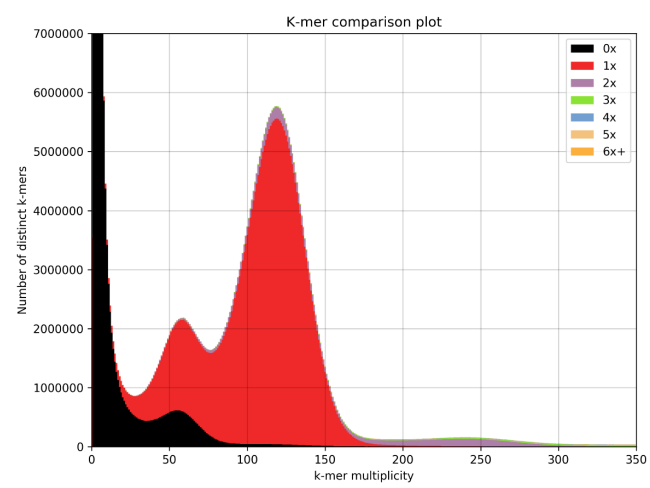

**B**

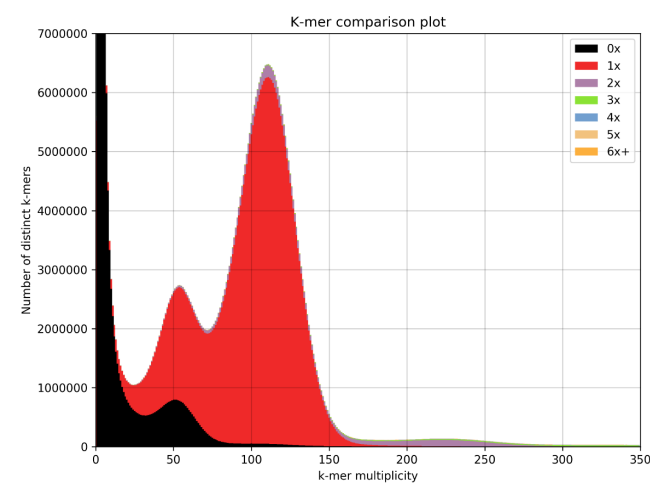

**C**

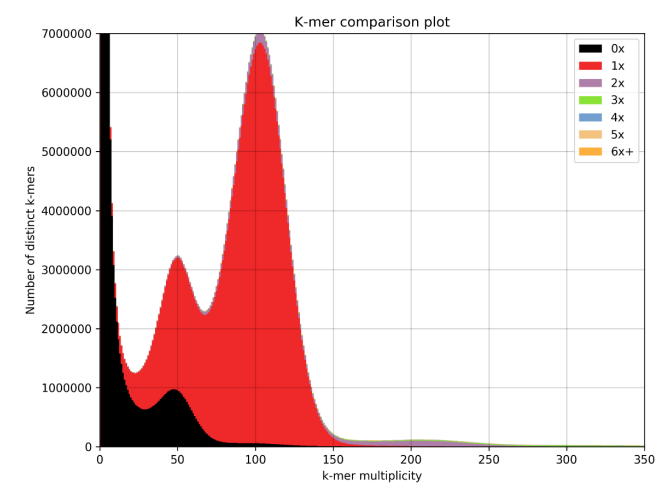

Supplement: jkad060_Supplementary_Data [file jkad060_supplementary_data.zip › Figure_S2_G3-2023-404103.pdf]

A

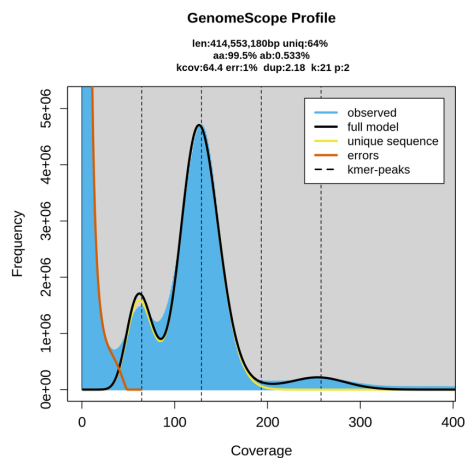

B

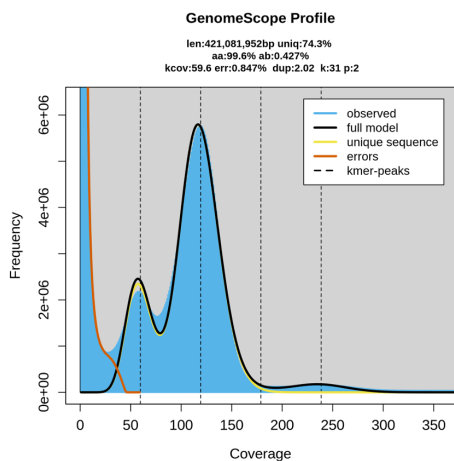

C

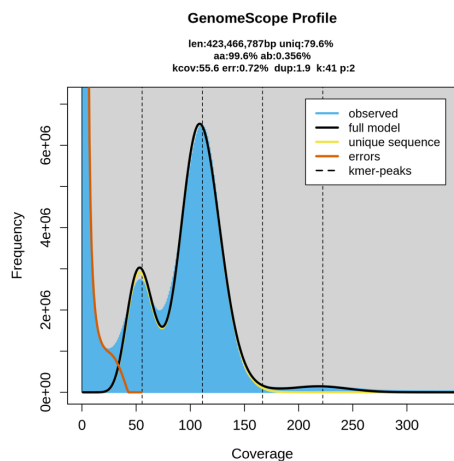

D

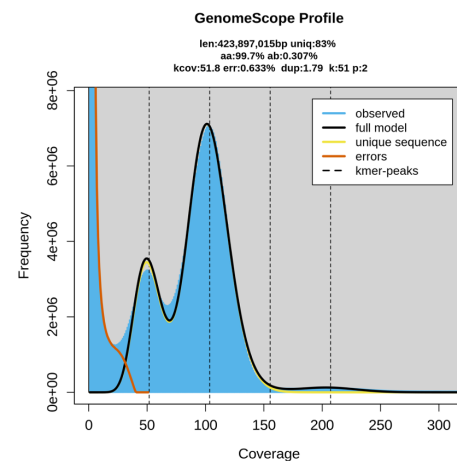

E

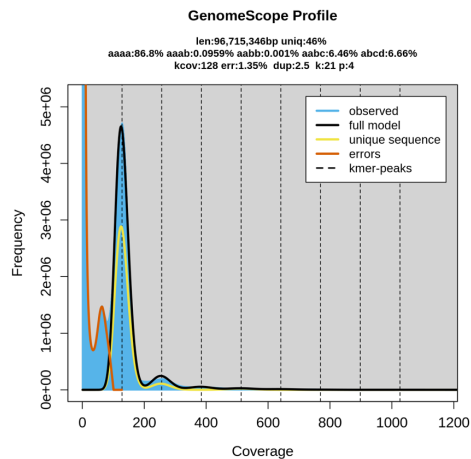

F

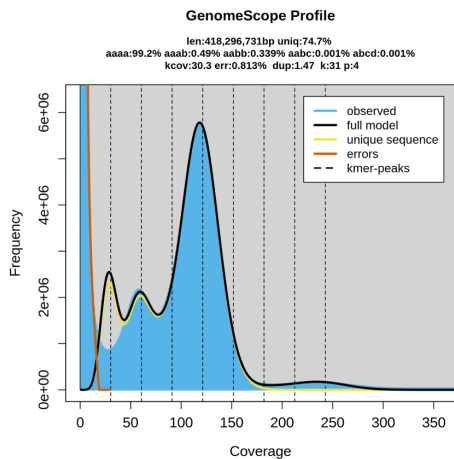

G

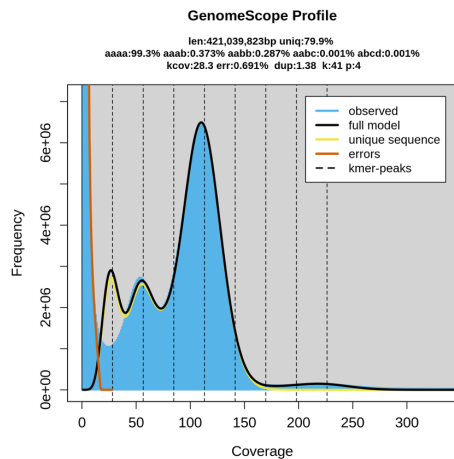

H

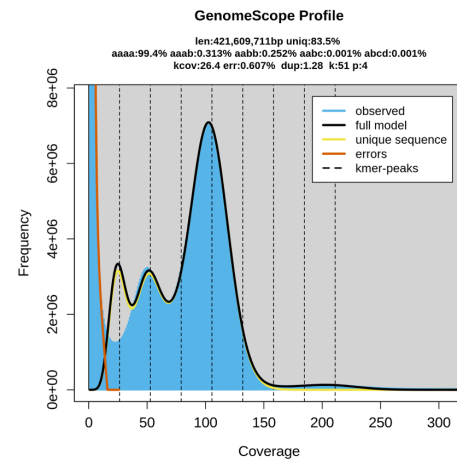

Supplement: jkad060_Supplementary_Data [file jkad060_supplementary_data.zip › Figure_S3_G3-2023-404103.pdf]

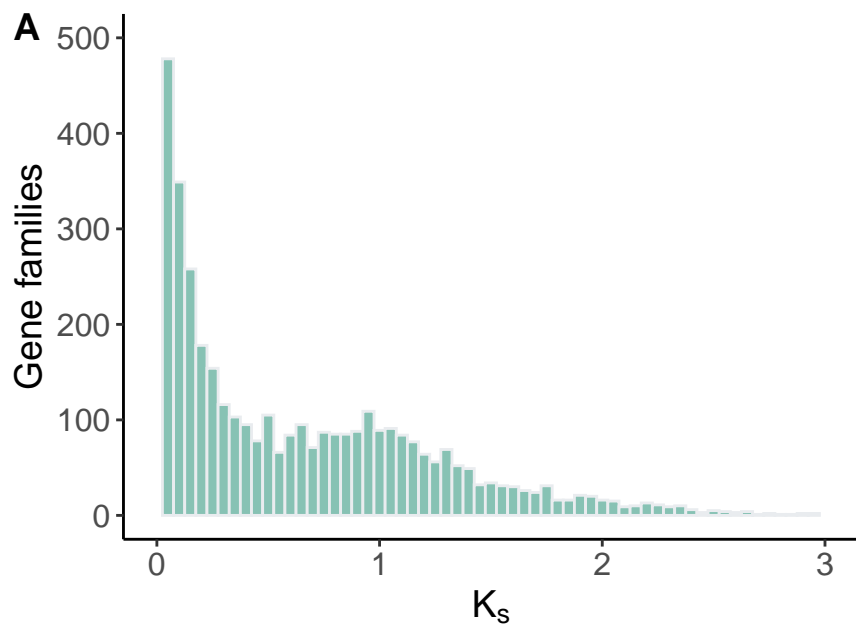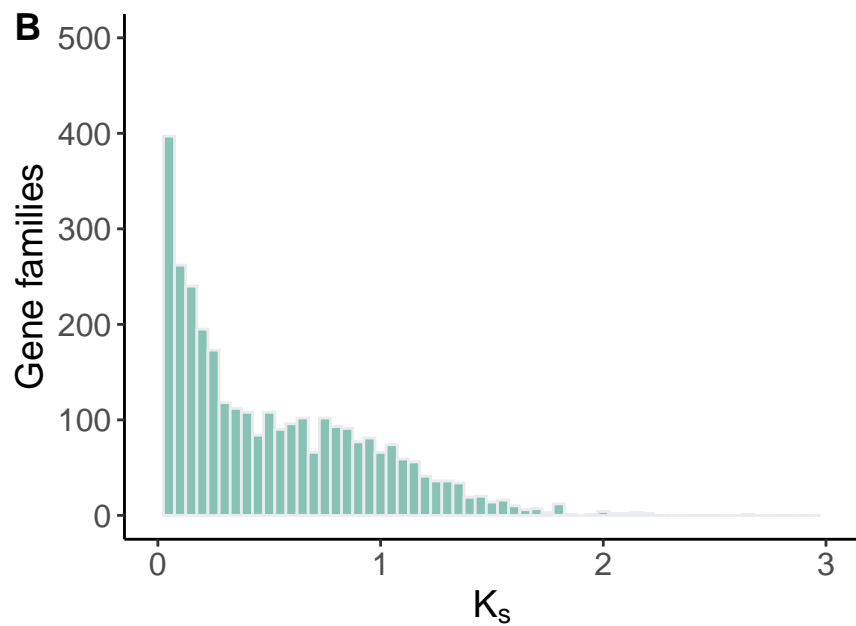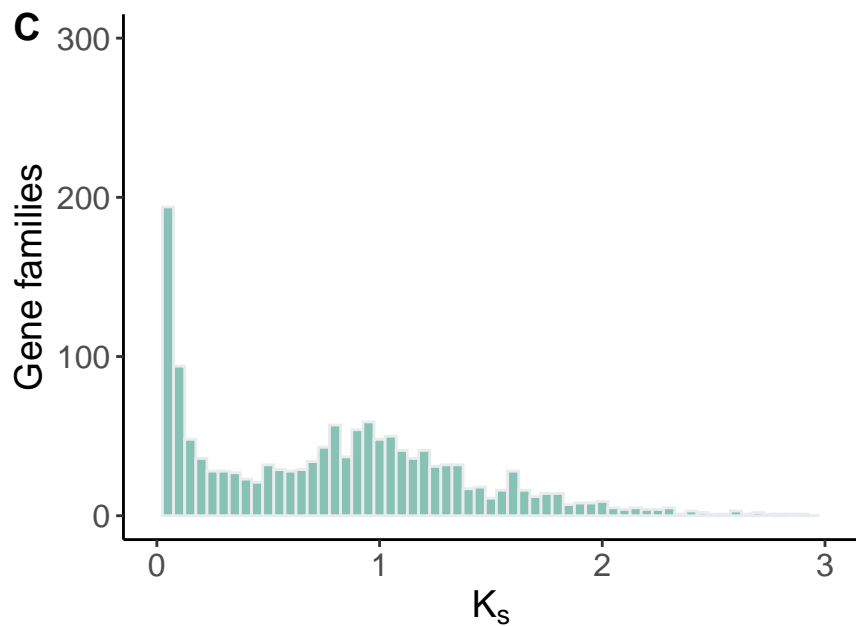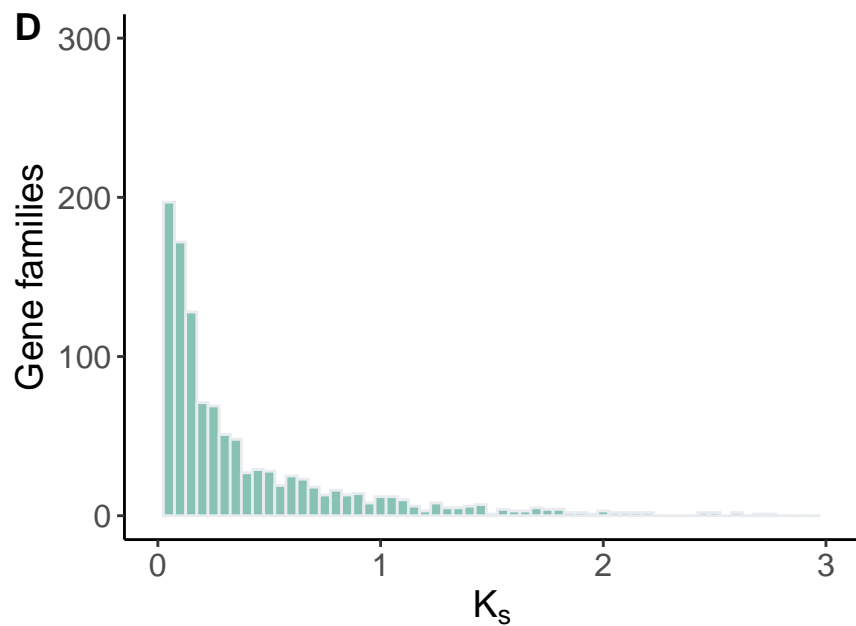

Supplement: jkad060_Supplementary_Data [file jkad060_supplementary_data.zip › Figure_S4_G3-2023-404103.pdf]

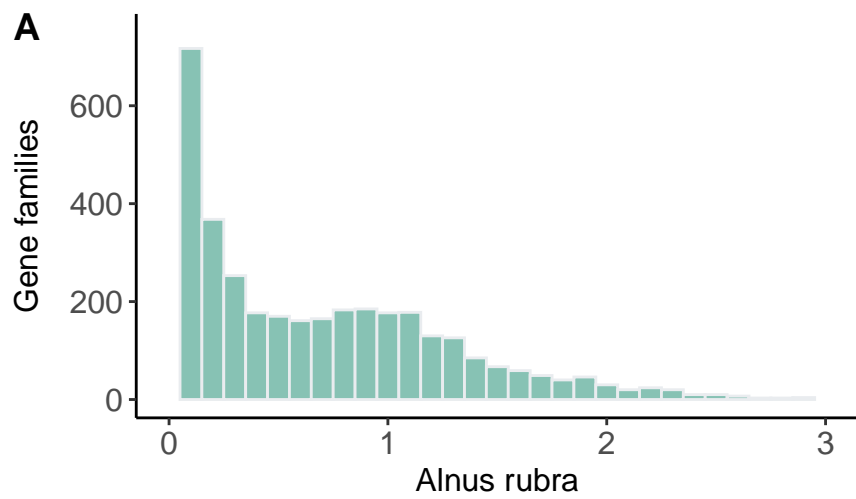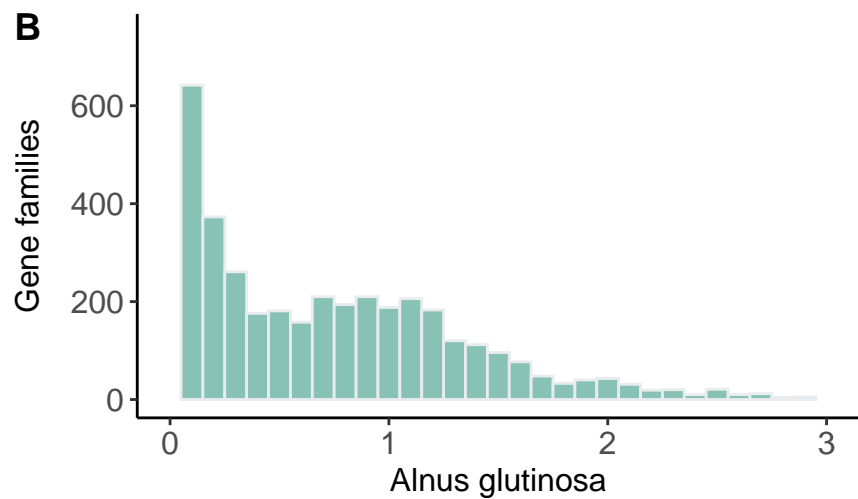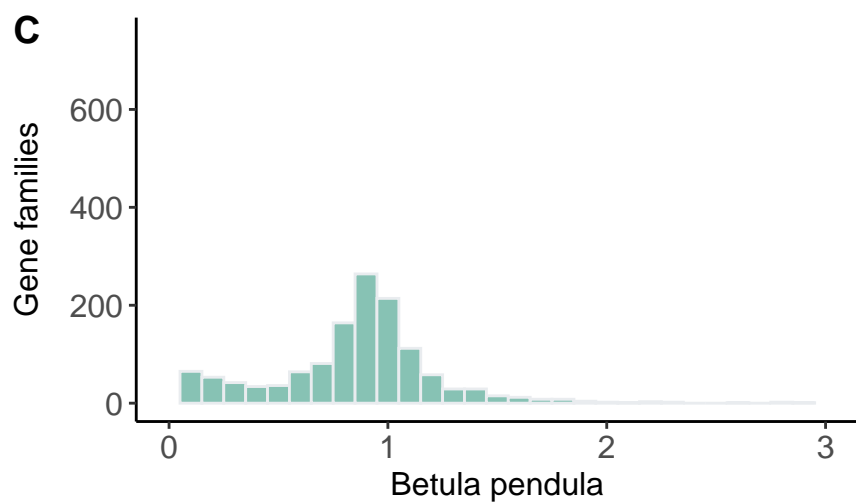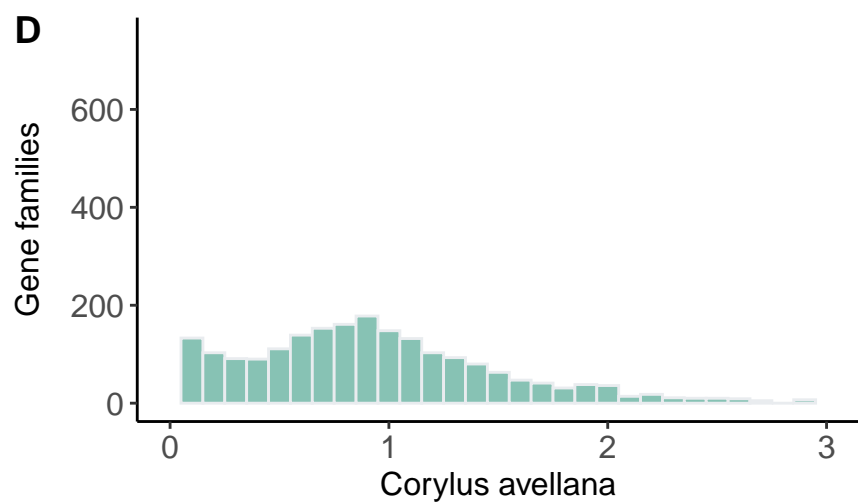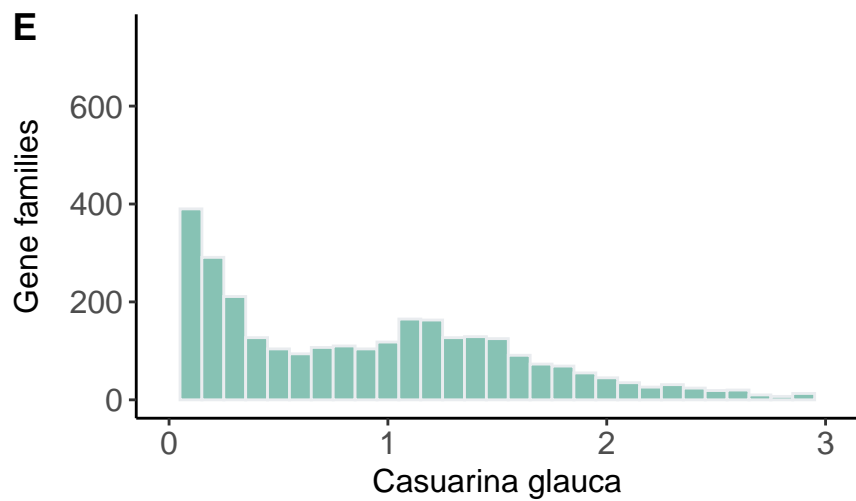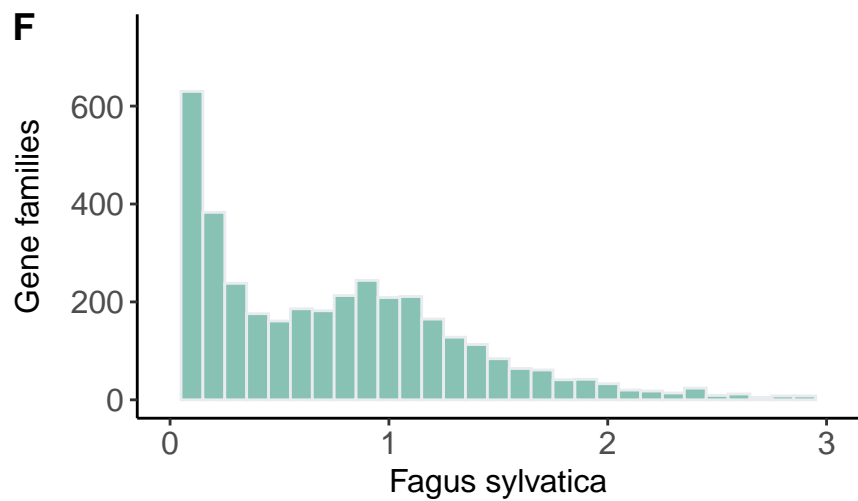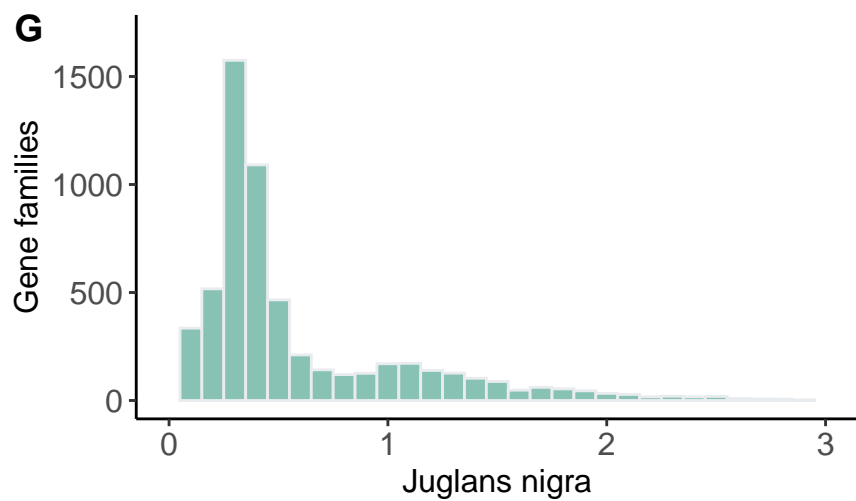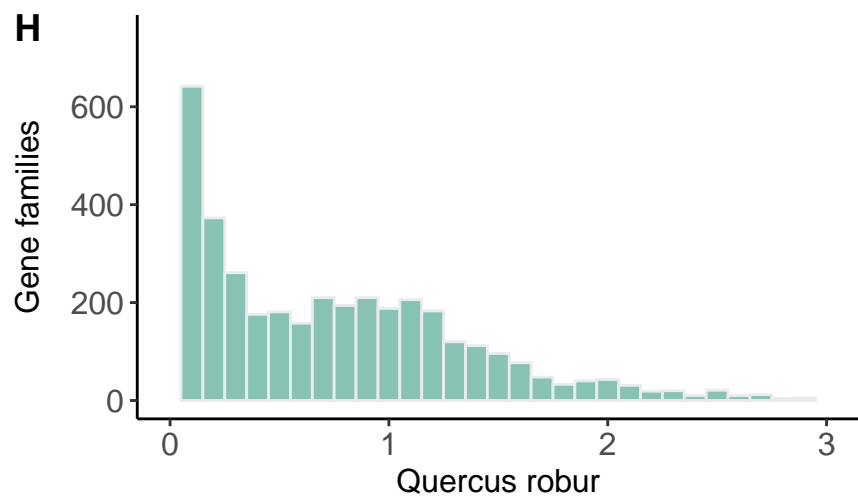

Supplement: jkad060_Supplementary_Data [file jkad060_supplementary_data.zip › Figure_S5_G3-2023-404103.pdf]
